# MUSCLE WEAKNESS IS ASSOCIATED WITH DISABILITY AND CHRONIC MULTIMORBIDITY IN MIDDLE-AGED AND OLDER AMERICANS

**DOI:** 10.1093/geroni/igz038.1155

**Published:** 2019-11-08

**Authors:** Mark Peterson, Jessica Faul

**Affiliations:** University of Michigan, Ann Arbor, Michigan, United States

## Abstract

Background: The objective of this study was to use nationally-representative data on Americans age 50+ to determine the association between grip strength and inflammation as independent predictors of incident disability, chronic multimorbidity and dementia. Methods: Older adults (n=12,618) from the 2006-2008 waves of the Health and Retirement Study with 8-years of follow-up were included. Longitudinal modeling was performed to examine the association between baseline grip strength (normalized to body mass: NGS) and high sensitivity c-reactive protein (hs-CRP) (≥3.0 mg/L) with incident physical disabilities (i.e., ≥2 limitations to activities of daily living), chronic multimorbidity (≥2 of chronic conditions), and dementia. Results: The odds of incident disability were 1.28 (95% CI: 1.19-1.37) and 1.27 (95% CI: 1.21-1.36) for men and women respectively, for each 0.05-unit lower NGS. The odds of incident chronic multimorbidity were 1.22 (95% CI: 1.06-1.18) and 1.12 (95% CI: 1.06-1.17) for men and women respectively for each 0.05-unit lower NGS. The odds of incident dementia were 1.10 for men (95% CI: 1.02-1.20) for each 0.05-unit lower NGS, but there was no significant effect for women. Elevated hs-CRP was only associated with chronic multimorbidity among women (OR=1.60; 95%CI: 1.26-2.02). Conclusions: Our findings indicate a robust inverse association between NGS and disability and chronic, multimorbidity in older men and women, and dementia in men. Elevated hs-CRP was only associated with chronic multimorbidity among women. Healthcare providers should implement measures of handgrip strength in routine health assessments and discuss the potential dangers of weakness and interventions to improve strength with their patients.

